# Genome-wide insights into the evolutionary history of conserved photosynthetic NDH-1 in cyanobacteria

**DOI:** 10.3389/fpls.2025.1561629

**Published:** 2025-04-15

**Authors:** Xiaoqin Pang, Yuanyuan Jiang, Jie Yu, Zhaoxing Ran, Weimin Ma

**Affiliations:** College of Life Sciences, Shanghai Normal University, Shanghai, China

**Keywords:** cyanobacteria, conserved photosynthetic NDH-1, OPS subunits, evolutionary history, cyclic electron transfer

## Abstract

The integration of novel components into functional multi-subunit protein complexes is a key evolutionary strategy for enhancing stability, activity, and adaptation to oxidative stress. This is exemplified by the evolution of the conserved photosynthetic NDH-1 (cpNDH-1) complex, though its precise evolutionary history remains unresolved. In this study, we constructed a time-calibrated phylogenetic tree of cyanobacteria to trace the evolutionary trajectory of cpNDH-1. By mapping the orthologous of oxygenic photosynthesis-specific (OPS) subunits onto this tree, we found that the cpNDH-1 complex progressively acquired OPS subunits. Specifically, during the transition from non-photosynthetic to thylakoid-less photosynthetic cyanobacteria, cpNDH-1 incorporated OPS subunits NdhM, NdhN, NdhO, NdhP, and NdhS. Subsequently, NdhL, NdhQ, and NdhV were added as thylakoid-bearing photosynthetic cyanobacteria evolved. Our analysis reveals that the emergence of oxygenic photosynthesis was closely linked with the progressive incorporation of OPS subunits into cpNDH-1. We propose a two-step model for the evolution of these subunits, identifying potential driving factors behind this process. Genome-wide sequence analysis and structural predications further suggest that the OPS cpNDH-1 genes either evolved *de novo* or arose from modifications of existing genes. Collectively, these findings provide a robust framework for understanding the evolutionary emergence of OPS subunits in cyanobacterial cpNDH-1, underscoring the acquisition of new subunits as a critical adaptation to oxidative environments during the evolution of oxygenic photosynthesis.

## Introduction

Approximately 2.4 billion years ago, cyanobacteria revolutionized Earth’s atmosphere by initiating oxygenic photosynthesis, which led to the Great Oxidation Event (GOE) and significantly altered the planet’s environment (Bekker et al., 2004). The rise of atmospheric O_2_ exposed cyanobacteria to unprecedented oxidative stress, driving evolutionary adaptations that were essential for their survival and the continued evolution of oxygenic photosynthesis (Fischer et al., 2016; Khademian and Imlay, 2021). In this challenging environment, maintaining the stability and functionality of multi-subunit protein complexes became crucial. The addition of subunits is a well-documented mechanism by which such complexes adapt to oxidative stress (Marsh and Teichmann, 2015; Urano et al., 2016). For example, prior to the rise of oxygen, ribulose-1,5-bisphosphate carboxylase/oxygenase (Rubisco) acquired an accessory subunit to enhance its specificity for CO_2_ over O_2_ (Schulz et al., 2022). Similarly, superoxide dismutase diversified into four distinct forms, each with different catalytic metals, to better manage the oxidative challenges posed by elevated O_2_ levels (Boden et al., 2021). Thus, the gain of subunits represents a fundamental evolutionary strategy that enhanced the functionality of multi-subunit protein complexes, enabling cyanobacteria to adapt to oxidative stress.

In cyanobacteria, the photosynthetic NDH-1 (pNDH-1) complex is a critical multi-subunit enzyme involved in cyclic electron transport around photosystem I (PSI CET), a process that reduces reactive oxygen species (ROS) production by providing additional ATP for photosynthetic carbon assimilation. This activity is essential for efficient photosynthesis and serves as a key antioxidant mechanism (Zhang et al., 2020; Miller et al., 2021). Notably, pNDH-1 has undergone evolutionary diversification, with four distinct complex types—pNDH-1L, pNDH-1L’, pNDH-1MS, and pNDH-1MS’ (Battchikova et al., 2011a; Ma and Ogawa, 2015; Laughlin et al., 2020)—each fulfilling a common role in PSI CET (Bernát et al., 2011). The conserved pNDH-1L form (hereafter referred to as cpNDH-1) is considered the ancestral form and the precursor to the other three types (Peltier et al., 2016).

The structure of cyanobacterial cpNDH-1 is L-shaped (Zhang et al., 2020), a feature shared with its archaeal group 4 membrane-bound [NiFe] hydrogenase (Yu et al., 2018) and its bacterial homolog, respiratory complex I (Baradaran et al., 2013). This architecture enables the efficient coupling of electron transfer, mediated by a hydrophilic arm extending into the cytoplasm, with proton translocation carried out by the membrane-embedded arm. The structural stability of cpNDH-1 is crucial for this coupling and, consequently, for PSI CET activity. Unlike its ancestral and homologous counterparts, cyanobacterial cpNDH-1 incorporates eight additional subunits, termed oxygenic photosynthesis-specific (OPS) subunits (NdhL to NdhQ, NdhS, and NdhV), which are critical for the structural integrity and function of the complex (Ma and Ogawa, 2015; Zhang et al., 2020). These OPS subunits are involved in various processes, such as electron donor binding (NdhO, NdhS and NdhV), assembly of the hydrophilic arm (NdhM and NdhN), stabilization of the membrane arm (NdhP and NdhQ), and formation of the plastoquinone (PQ) channel (NdhL). The absence of these OPS subunits destabilizes cpNDH-1 (Battchikova et al., 2005, 2011b; Zhang et al., 2014; Zhao et al., 2014, 2015; Gao et al., 2016; He et al., 2016; He and Mi, 2016), resulting in reduced PSI CET activity, compromised ATP production, and a disrupted ATP/NADPH ratio, which in turn impairs carbon fixation (Zhang et al., 2020; Zheng et al., 2025). Under aerobic stress conditions, this disruption leads to increased ROS production.

In 2007, a study of 17 cyanobacterial strains focused on the evolutionary history of OPS subunits NdhL, NdhM, NdhN, and NdhO (Ogawa and Mi, 2007). However, the incomplete identification of other OPS subunits and the lack of comprehensive evolutionary data at that time limited our understanding of their evolution. These OPS subunits are generally small and structurally simple (**Supplementary Figure S1**). Moreover, with the exception of NdhV, they lack unique domains, with NdhV belonging to the DUF2996 domain-containing protein group. Despite significant progress in understanding cpNDH-1 evolution (Peltier et al., 2016; Kato et al., 2021; Yu et al., 2021, 2022), the precise evolutionary trajectory of these OPS subunits remains unresolved.

Recent discoveries of sister groups to cyanobacteria, including the non-photosynthetic species like *Candidatus Melainabacteria* and *Candidatus Sericytochromatia* (Di Rienzi et al., 2013; Soo et al., 2017), as well as new thylakoid-less species such as *Candidatus Aurora vandensis* and *Anthocerotibacter panamensis* (Grettenberger et al., 2020; Rahmatpour et al., 2021), have provided critical insights into the early evolution of cyanobacteria. These findings, along with expanding genomic data, offer an unparalleled opportunity to further elucidate the evolutionary history of OPS subunits. In this study, we constructed a time-calibrated phylogenetic tree of cyanobacteria to trace the evolution of cpNDH-1. Our analysis reveals that cpNDH-1 progressively acquired OPS subunits throughout cyanobacterial evolution, coinciding with the emergence and diversification of oxygenic photosynthesis. Based on these findings, we propose a two-step model for the acquisition of OPS subunits, suggesting that their incorporation was crucial for the adaptation of cpNDH-1 to oxygenic photosynthesis. Furthermore, we investigate potential OPS homologs in both non-photosynthetic and thylakoid-less photosynthetic cyanobacteria through protein sequence and structural analyses, providing new insights into the origins of OPS genes. Together, our findings illuminate the timing and mechanisms underlying the evolution of OPS subunits in cpNDH-1.

## Materials and methods

### Time-calibrated phylogenetic analysis

A time-calibrated phylogenetic tree for the phylum Cyanobacteria was constructed using BEAST v1.10.4 (Drummond and Rambaut, 2007) based on 16S rDNA sequences. These sequences were retrieved from the National Center for Biotechnology Information (NCBI, https://www.ncbi.nlm.nih.gov) and Cydrasil 3 (https://www.cydrasil.org) (Roush et al., 2021). The species names and their corresponding 16S rDNA sequences used in this study are provided in **Supplementary Data 1**. Sequence alignment and gap removal were performed using MUSCLE (Edgar, 2004). The original phylogenetic tree employed for time calibration is presented in **Supplementary Figure S2**.

Three calibration points, previously established in the literature, were applied (Blank and Sánchez-Baracaldo, 2010; Sánchez-Baracaldo et al., 2014). The prior distribution for *Nostocales* was modeled as a normal distribution with a mean of 2,250 million years ago (Mya) and a standard deviation of 100, truncated between 2,100 and 2,450 Mya (Tomitani et al., 2006; Sánchez-Baracaldo et al., 2014). For *Pleurocapsales*, the prior distribution was set as a normal distribution with a mean of 2,050 Mya and a standard deviation 200, truncated between 1,700 and 2,450 Mya (Sánchez-Baracaldo et al., 2014). The cyanobacterial root age was constrained to a uniform distribution between 2,320 and 2,700 Mya (Bekker et al., 2004).

The analysis employed a Yule process speciation model with an uncorrelated log-normal relaxed clock model. A total of 50 million iterations were run, with sampling every 5,000 iterations. Convergence and posterior distributions were assessed using Tracer v1.6, and the maximum clade credibility tree was generated with TreeAnnotator v1.10.4 from the BEAST package. The resulting phylogenetic tree was visualized and edited using FigTree v1.4.4 (http://tree.bio.ed.ac.uk/software/figtree).

### Conserved *ndh* genes analysis

The analysis of conserved *ndh* gene was conducted across a range of cyanobacterial species, including non-photosynthetic cyanobacteria *Candidatus Melainabacteria* (*Melaina*) and *Candidatus Sericytochromatia* (*Seri*); thylakoid-less cyanobacteria *Anthocerotibacter panamensis* (*A. pana*) and *Gloeobacter violaceus* PCC7421 (*Gloeo*7421); and thylakoid-bearing cyanobacteria *Synechococcus* sp. PCC7336 (*Syn*PCC7336), *Synechococcus* sp. JA-3-3Ab (*Syn*JA33Ab), *Synechococcus* sp. PCC7502 (*Syn*PCC7502), *Pseudanabaena* sp. PCC7367 (*Pseud*7367), *Synechocystis* sp. PCC6803 (*Syn*PCC6803), *Thermosynechococcus elongatus* BP-1 (*T. elongatus*), *Synechococcus* sp. PCC7942 (*Syn*PCC7942), *Synechococcus* sp. WH5701 (*Syn*WH5701), *Synechococcus* sp. WH8102 (*Syn*WH8102), and *Prochlorococcus marinus* CCMP1375 (*Pro*CCMP1375). These species were selected based on the availability of complete or nearly complete genome sequences. The GenBank accession numbers for all species are provided in **Supplementary Data 2**.

Orthologous protein sequences corresponding to conserved subunits of the cpNDH-1 complex from *Synechocystis* sp. PCC6803 were identified in the aforementioned species using BLASTP searches on NCBI. The top hits for each sequence were verified through reciprocal BLASTP against *Synechocystis* sp. PCC6803. The NCBI reference sequence accession numbers for these proteins are listed in **Supplementary Data 2**.

The genomic positions of the *ndh* genes for each species were retrieved to construct the *ndh* gene cluster architecture. Comparative structural analyses of these *ndh* gene clusters were visualized by ChiPlot (https://www.chiplot.online).

### Sequence analysis of OPS cpNDH-1 subunits

To investigate the evolutionary history of OPS cpNDH-1 subunits, we used the protein sequence of OPS cpNDH-1 subunits from *Synechocystis* sp. PCC6803 to identify homologous sequences in the aforementioned cyanobacterial species via BLASTP searches, applying a stringent *E*-value threshold of 1.0 × 10^-6^. The sequence data and their similarities are provided in **Supplementary Data 3**. To confirm the evolutionary history, phylogenetic trees of each OPS cpNDH-1 subunit were constructed. Detailed sequences information is available in **Supplementary Data 4**.

To trace the origins of OPS cpNDH-1 subunits, we employed two approaches: one based on sequence information and the other on structural data. In the sequence-based approach, we identified potential homologous fragments by comparing the amino acid sequences of OPS cpNDH-1 subunits to sequences from non-photosynthetic and thylakoid-less cyanobacteria in local nucleotide databases using TBLASTN. Protein sequences of OPS subunits from cyanobacterial cpNDH-1 served as templates. To enable a broader search, the TBLASTN expectation value threshold was set to 10. The raw alignment data are available in **Supplementary Data 5**. In the structure-based approach, the AlphaFold server (Abramson et al., 2024) was used to predict the structures of the earliest OPS subunits. These predicted structures were then employed as templates to identify distant orthologs using Foldseek (van Kempen et al., 2024).

### Data availability

All data supporting the findings of this study are included in the main text and **Supplementary Information** files.

## Results and discussion

### Time-calibrated phylogenetic tree reveals cyanobacterial evolution

**Figure 1:**
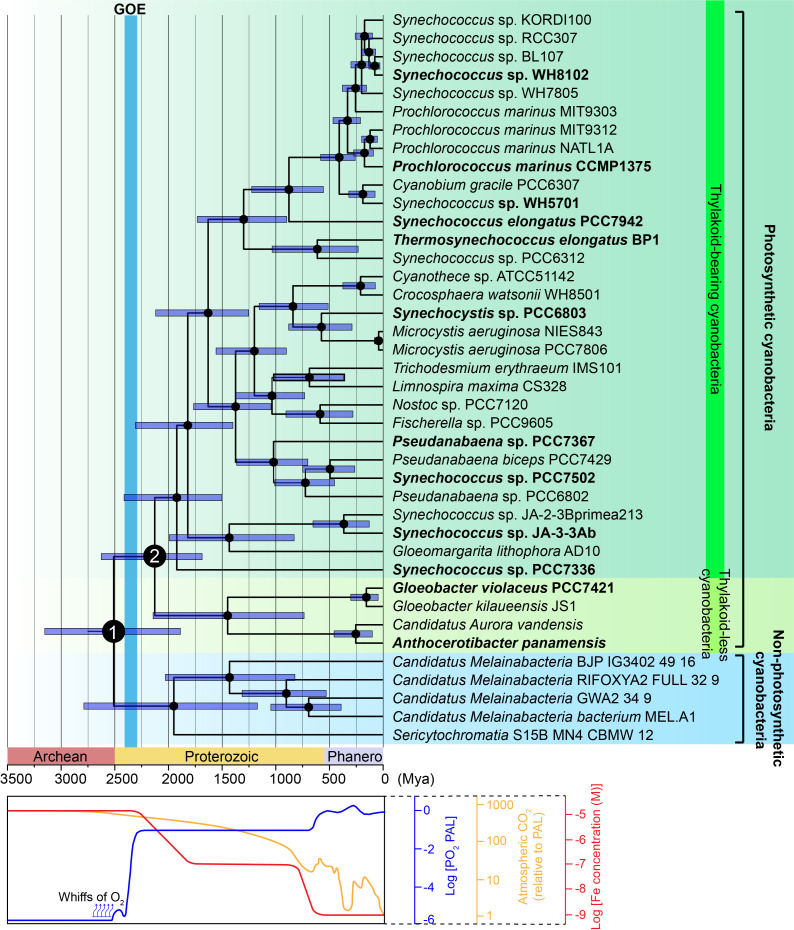
Time-calibrated phylogenic tree of the phylum Cyanobacteria. The phylogenetic tree was generated using Bayesian analysis of aligned 16S rDNA sequences. (Upper panel) Bayesian relaxed molecular clock analyses, implemented in BEAST, were used to estimate divergence times. Three calibrations points were applied (see Materials and Methods). Dark blue bars represent the posterior 95% confidence intervals for node ages. Non-photosynthetic cyanobacteria (blue background) form the sister group to photosynthetic cyanobacteria. Photosynthetic cyanobacteria are subdivided into the class *Gloeobacteria* (thylakoid-less, chartreuse background) and crown cyanobacteria (thylakoid-bearing, green background) based on the presence or absence of thylakoid membrane. Node 1 marks the divergence between non-photosynthetic and photosynthetic cyanobacteria, while Node 2 represents the divergence between thylakoid-less *Gloeobacteria* and thylakoid-bearing cyanobacteria. The Great Oxidation Event (light blue bar) occurred approximately 2,400 Mya. Photosynthetic cyanobacteria species selected for detailed analysis are highlighted in bold. The original phylogenetic tree used for time calibration is presented in **Supplementary Figure S2**. (Lower panel) Timeline of major evolutionary and geochemical transitions. Changes in atmospheric O_2_ (blue line), CO_2_ (yellow line), and Fe (red line) concentrations were adapted from He et al. (2021); Flamholz and Shih (2020), and Anbar (2008), respectively.

Recent advancements in genome and metagenome sequencing have significantly expanded our understanding of cyanobacterial evolution and the diversification of their multi-subunit protein complexes (Di Rienzi et al., 2013; Soo et al., 2017; Grettenberger et al., 2020; Rahmatpour et al., 2021). To explore the evolutionary origins of cpNDH-1 within the phylum Cyanobacteria, we reconstructed a time-calibrated phylogenetic tree (**Figure 1**). This analysis underscores key evolutionary transitions within cyanobacteria.

The base of the tree is occupied by non-photosynthetic cyanobacteria (indicated by the blue background in **Figure 1**), including *Candidatus Sericytochromatia* and *Candidatus Melainabacteria*, which are regarded as the closest living relatives of photosynthetic cyanobacteria (Blankenship, 2017). The divergence between non-photosynthetic and photosynthetic cyanobacteria is estimated to have occurred around 2,500 million years ago (Mya) (Node 1, **Figure 1**).

Within photosynthetic cyanobacteria, the class *Gloeobacteria*, comprising *Candidatus Aurora vandensis*, *Anthocerotibacter panamensis*, and the genus *Gloeobacter*, is characterized by the absence of thylakoid membranes, positioning it as a critical lineage for understanding the early stages of cyanobacterial evolution (Rahmatpour et al., 2021) (indicated by the chartreuse background in **Figure 1**). Thylakoid-bearing cyanobacteria, in which thylakoid membranes evolved, form a distinct, more derived group (indicated by the green background in **Figure 1**). The divergence between thylakoid-less and thylakoid-bearing cyanobacteria is estimated to have occurred between 2,500 and 2,000 Mya (Node 2, **Figure 1**). This trajectory—spanning from non-photosynthetic to photosynthetic thylakoid-less cyanobacteria, and ultimately to photosynthetic thylakoid-bearing cyanobacteria—aligns with prevailing models of cyanobacterial evolution (Garcia-Pichel et al., 2020; Sánchez-Baracaldo and Cardona, 2020; Sánchez-Baracaldo et al., 2022). However, discrepancies persist regarding the placement of *Gloeomargarita lithophora* AD10 and *Pseudanabaena* (Fournier et al., 2021; Strunecký et al., 2023).

**Figure 2:**
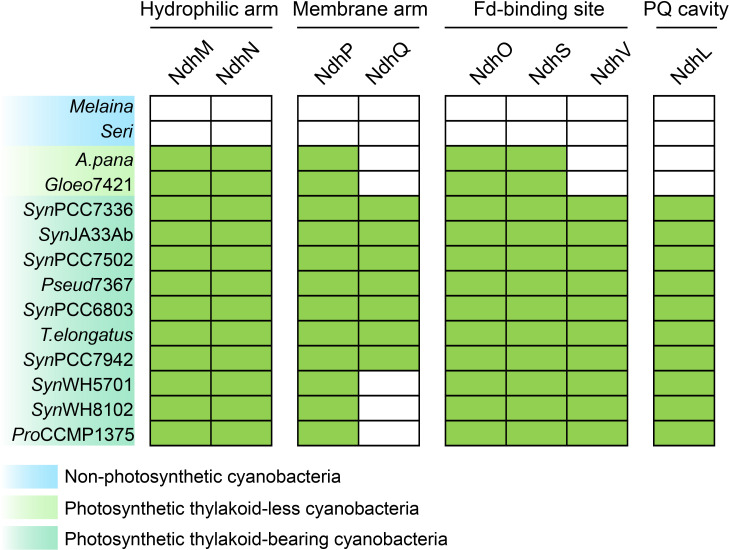
Distribution of OPS cpNDH-1 subunits in non-photosynthetic and photosynthetic cyanobacteria. The presence and absence of homologs of OPS cpNDH-1 subunits, identified through BLASTP searches, are mapped onto the evolutionary tree of selected cyanobacteria shown in **Figure 1**. Filled boxes denote the presence of genes, while empty boxes indicate their absence. The sequence information and their similarity are provided in **Supplementary Data 3**. Strain abbreviations are provided in the Materials and Methods section.

In non-photosynthetic cyanobacteria, the genes for the 11 conserved subunits of cpNDH-1 (NdhA-NdhK) are already present (**Supplementary Figure S3**), suggesting that the core components of cpNDH-1 evolved in the last common ancestor of photosynthetic cyanobacteria. However, genes encoding the OPS subunits are absent in these non-photosynthetic cyanobacteria (**Figure 2**), supporting the hypothesis that the acquisition of these subunits coincided with the transition to photosynthetic functions such as PSI CET in cyanobacteria. Future investigations will focus on pinpointing the timing and mechanisms by which these OPS subunits were integrated into cpNDH-1.

### The emergence of OPS cpNDH-1 subunits

To explore the evolutionary integration of OPS subunits into cpNDH-1, we conducted BLASTP searches to identify orthologous genes, followed by mapping these data onto the reconstructed cyanobacterial phylogeny. Phylogenetic trees of these OPS subunits were also constructed (**Supplementary Figure S4**), providing insights into the progressive acquisition of these subunits throughout cyanobacterial evolution (**Figure 2**).

Following the emergence of photosynthetic thylakoid-less cyanobacteria (Node 1, **Figure 1**), five OPS subunits were integrated to cpNDH-1 (**Figure 2**; **Supplementary Figure S5**). These include NdhM and NdhN in the hydrophilic arm, NdhP in the membrane arm, and NdhO and NdhS near the ferredoxin (Fd)-binding site (Laughlin et al., 2019; Schuller et al., 2019; Pan et al., 2020; Zhang et al., 2020) (**Supplementary Figure S1**). These subunits are essential for stabilizing cpNDH-1, as demonstrated in *Synechocystis* sp. PCC6803, where the absence of NdhM, NdhN, or NdhP destabilizes cpNDH-1, while NdhO and NdhS are critical for Fd binding (Battchikova et al., 2011b; Zhang et al., 2014; Zhao et al., 2014; He and Mi, 2016; He et al., 2016; He and Mi, 2016).

As thylakoid-bearing cyanobacteria, such as *Synechococcus* sp. PCC7336, emerged (Node 2, **Figure 1**), additional OPS subunits were integrated into cpNDH-1 (Laughlin et al., 2019; Schuller et al., 2019; Pan et al., 2020; Zhang et al., 2020). These included NdhV at the Fd-binding site, enhancing Fd-binding efficiency (Gao et al., 2016), and NdhQ in the membrane arm (**Figure 2**; **Supplementary Figures S1**, **S5**), which works synergistically with NdhP to stabilize the cpNDH-1 structure (Zhao et al., 2015). The incorporation of NdhL further reinforced the PQ cavity, ensuring efficient PSI CET (Mi et al., 1992; Zhang et al., 2020).

**Figure 3:**
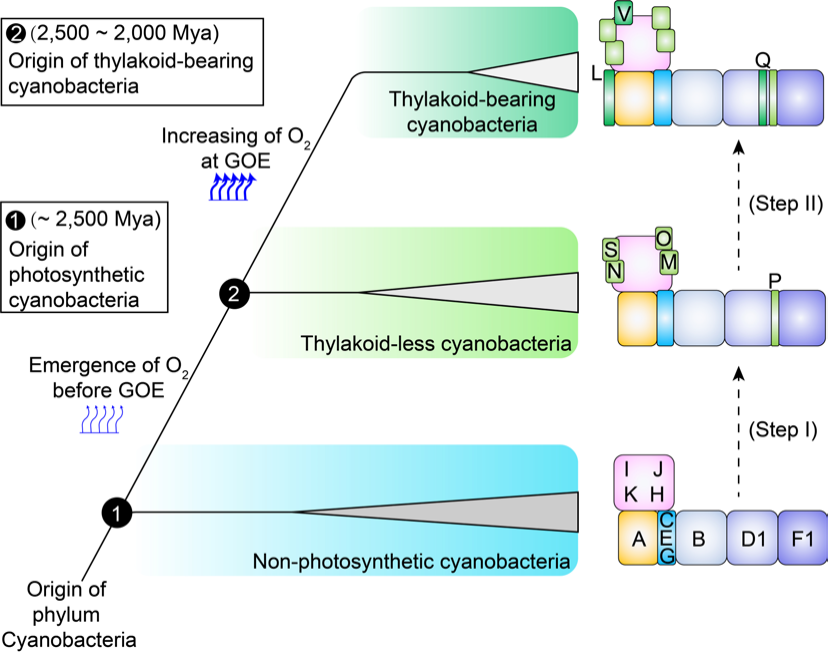
A two-step schematic model illustrating the evolutionary history of OPS subunits in cyanobacterial cpNDH-1. In non-photosynthetic cyanobacteria, 11 conserved *ndh* genes are present. The acquisition of OPS cpNDH-1 genes occurred in two distinct evolutionary phases during the transition to photosynthetic cyanobacteria. Step I: During the evolution from non-photosynthetic to photosynthetic cyanobacteria, several OPS subunits (NdhM to NdhP, NdhS) were incorporated, enhancing the stability and activity of cpNDH-1. Step II: As thylakoid-less *Gloeobacteria* evolved into crown cyanobacteria, additional OPS subunits (NdhL, NdhQ and NdhV) were acquired, further stabilizing and optimizing cpNDH-1 activity. Subunits are represented by black text, labeled according to their designations in cpNDH-1. The emergence and rise of oxygen levels are depicted with thin and thick arrows, respectively.

The acquisition of OPS subunits aligns with two key transitions in cyanobacterial photosynthesis, providing a potential evolutionary scenario for these subunits (**Figure 3**). During the shift from non-photosynthetic to thylakoid-less cyanobacteria (Step I, **Figure 3**), early oxygenic photosynthesis occurred a geochemically constrained environment, resulting in slow oxygen evolution and intermittent oxygen generation (Rahmatpour et al., 2021), setting the stage prior to the GOE (**Figure 1**). Rising oxygen levels and ROS likely triggered oxidative stress, disrupting the stabilization of cpNDH-1. The recruitment of NdhM, NdhN, NdhP, and NdhS would have been critical for this adaptation, potentially ensuring the stabilization and functionality of cpNDH-1 in an increasingly oxidative environment.

As cyanobacteria transitioned to thylakoid-bearing species (Step II, **Figure 3**), the evolution of thylakoid membranes accelerated oxygenic photosynthesis, producing a large amount of oxygen (Rast et al., 2015), driving the GOE (Guéguen and Maréchal, 2022) (**Figure 1**). Thylakoid-less cyanobacterial species, such as *Anthocerotibacter panamensis* and *Gloeobacter violaceus* PCC7421, exhibited limited photosynthetic capacity (Rahmatpour et al., 2021; Raven and Sánchez-Baracaldo, 2021), with a cell doubling time of 3-8 days (Rippka et al., 1974; Rahmatpour et al., 2021). In contrast, the thylakoid-bearing cyanobacterium *Synechococcus* sp. PCC7336, the closest relative of thylakoid-less cyanobacteria (Tan et al., 2024), has a cell doubling time of approximately 1 day (Herrmann et al., 2021), while model species, such as *Synechocystis* sp. PCC6803, can divide within hours (Rahmatpour et al., 2021). This increased photosynthetic efficiency led to heightened ATP demand, further driving the need for cpNDH-1 stabilization.

To meet these demands, cpNDH-1 acquired NdhL, NdhQ, and NdhV, which are critical for maintaining high-efficiency PSI CET. The deletion of *ndhL* severely impairs PSI CET activity (Mi et al., 1992; Zhang et al., 2020), while the loss of *ndhV* disrupts Fd interaction (Gao et al., 2016; Zhang et al., 2020). These adaptations enabled cpNDH-1 to support its high levels of stabilization, potentially driving the evolution of robust oxygenic photosynthesis and carbon assimilation in thylakoid-bearing cyanobacteria. This analysis provides a hypothesis for the driving factors behind the evolution of OPS subunits, which will require further experimental validation.

### The evolutionary origin of OPS cpNDH-1 subunits

The evolutionary origins of OPS cpNDH-1 subunits provide valuable insights into their emergence and functional development. New genes can arise through various mechanisms, including *de novo* origination of genetic sequences or the modification of existing genes (Long et al., 2013).

**Figure 4:**
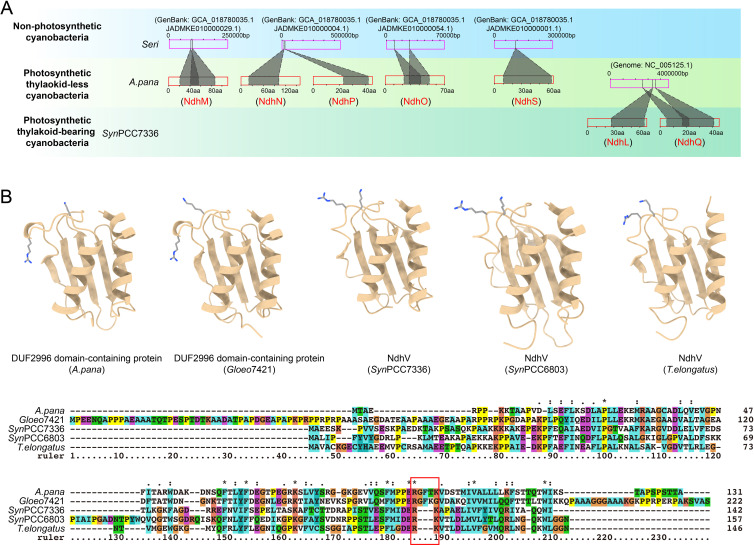
Gene origins of OPS subunits in cyanobacterial cpNDH-1. **(A)** TBLASTN and BLASTP analyses were performed to identify potential gene origins of OPS cpNDH-1 subunits. For each OPS cpNDH-1 subunit, the upper pink rectangle represents genomes or metagenome-assembled genomes, and the lower red rectangle represents the protein sequence of the OPS subunits as it first emerged in cyanobacteria. Shaded areas indicate regions of potential homology. The sequence data used for plotting are provided in **Supplementary Data 6**. Possible homologous fragments, rather than complete homologous sequences, identified in cyanobacteria with closely related evolutionary backgrounds suggest gene origination through *de novo* evolution. **(B)** Structure prediction (Upper panel) and sequence alignment (Lower panel) between DUF2996 domain-containing proteins from thylakoid-less cyanobacteria and NdhV from thylakoid-bearing cyanobacteria. Asterisks denote identical amino acids, while colons and periods indicate conserved and semi-conserved substitutions, respectively. The key residues involved in binding to the electron donor Fd in NdhV are highlighted by red box. The similar structures and changes in important amino acid sites suggest a hypothesis that NdhV may be generated by modifying existing gene of DUF2996 domain-containing proteins.

To investigate the origins of OPS cpNDH-1 genes, we performed TBLASTN searches to identify homologous gene fragments corresponding to specific OPS cpNDH-1 subunits in early-branching cyanobacteria. Our analysis revealed that NdhM, NdhN, NdhO, NdhP, and NdhS—subunits that first emerged in thylakoid-less cyanobacteria—are represented by only partial homologous fragments, rather than complete homologous sequences, in non-photosynthetic cyanobacteria of the class *Sericytochromatia* (**Figure 4A**; **Supplementary Data 5** and **6**). These results suggest that the genetic sequences giving rise to these OPS subunits were likely absent in non-photosynthetic cyanobacteria, implying that these subunits evolved through *de novo* origination.

Similarly, NdhL and NdhQ, which arose in thylakoid-bearing cyanobacteria, are also represented by only partial homologous fragments in thylakoid-less cyanobacteria (**Figure 4A**; **Supplementary Data 5** and **6**). This further supports the hypothesis that these subunits, too, may have evolved via *de novo* origination. Moreover, structure-based Foldseek analysis failed to identify any structurally similar homologs in cyanobacteria lacking OPS subunits, reinforcing the idea that these OPS subunits likely did not have ancestral counterparts in early cyanobacteria, further supporting their *de novo* evolution. However, the possibility remains that ancestral sequences of these OPS subunits existed, through verifying this would require more extensive genetic data from early-branching cyanobacteria lineages.

Interestingly, NdhV appears to have a distinct evolutionary origin compared to the other OPS subunits. NdhV contains a DUF2996 domain (Gao et al., 2016), and BLASTP and TBALSTN searches revealed no homologous fragments in thylakoid-less cyanobacteria (**Figure 2**; **Supplementary Data 5**). While DUF2996 domain-containing proteins are present in these cyanobacteria, their sequences show minimal similarity to NdhV from thylakoid-bearing cyanobacteria (**Supplementary Data 3**). Nevertheless, Foldseek analysis revealed significant structural similarity between the DUF2996 domain-containing proteins and NdhV (Sll0272) from *Synechocystis* sp. PCC 6803 (**Supplementary Figure S6**), which was further confirmed through structural alignments (**Figure 4B**). Sequence alignment of these structural homologs revealed that the two consecutive basic amino acids, critical for binding the electron donor Fd in NdhV (Zhang et al., 2020), are separated in the DUF2996 domain-containing proteins from thylakoid-less cyanobacteria (**Figure 4B**). Notably, this distance appears to shorten in the evolutionary trajectory of thylakoid-free cyanobacteria (indicated by the red box in **Figure 4B**). Collectively, these analyses suggest that NdhV may have evolved through the modification of an existing DUF2996 domain-containing proteins.

## Conclusions

In this study, we utilized extensive genomic data from cyanobacteria to investigate, for the first time, the evolutionary trajectory of OPS subunits in the cyanobacterial cpNDH-1 complex and proposed a possible evolutionary scenario for its development. Our findings suggest that, during the transition from non-photosynthetic to thylakoid-less photosynthetic cyanobacteria, the cpNDH-1 complex incorporated the OPS subunits NdhM, NdhN, NdhO, NdhP, and NdhS. Subsequently, during the shift from thylakoid-less to thylakoid-bearing cyanobacteria, additional OPS subunits—NdhL, NdhQ, and NdhV—were acquired. This proposed evolutionary trajectory not only reflects the adaptation of cyanobacteria to changing environmental stresses, but also offers a potential hypothesis for the driving forces behind the evolution of these subunits. Specifically, the rise of oxygen and the resulting oxidative stress, combined with the increased efficient oxygenic photosynthesis, likely intensified the demand for antioxidant defenses and PSI CET activity. Such evolutionary pressures may have driven the acquisition of OPS subunits to enhance the stability of cpNDH-1, a critical complex involved in these processes. Furthermore, protein sequence and structural analyses provide insights into the possible evolutionary origins of these OPS subunits. In conclusion, our findings highlight the potential role of oxygenic photosynthesis in shaping the evolution of cpNDH-1 in cyanobacteria and lay a groundwork for future studies exploring the functional and evolutionary significance of OPS subunits.

## Data Availability

The original contributions presented in the study are included in the article/**Supplementary Material**. Further inquiries can be directed to the corresponding authors.
