# Supplementary material for: Genome-wide insights into the evolutionary history of conserved photosynthetic NDH-1 in cyanobacteria
Source: Front Plant Sci. 2025 Apr 15;16:1561629. doi: 10.3389/fpls.2025.1561629 (PMC12038448; doi:10.3389/fpls.2025.1561629)
Supplement: Supplementary file 1 [file DataSheet1.pdf]

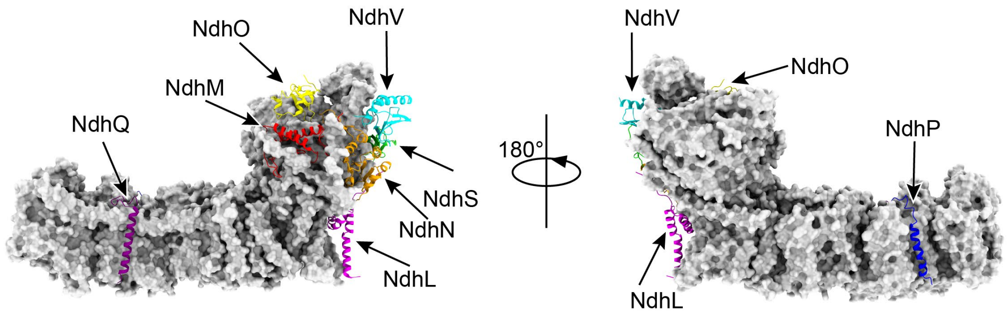

SUPPLEMENTARY FIGURE S1. Localization of OPS subunits within the cyanobacterial cpNDH-1 complex. The overall structure of cyanobacterial cpNDH-1 is shown in surface representation, with OPS subunits highlighted in ribbon format. The structure of cpNDH-1 is based on PDB entry 6L7O.

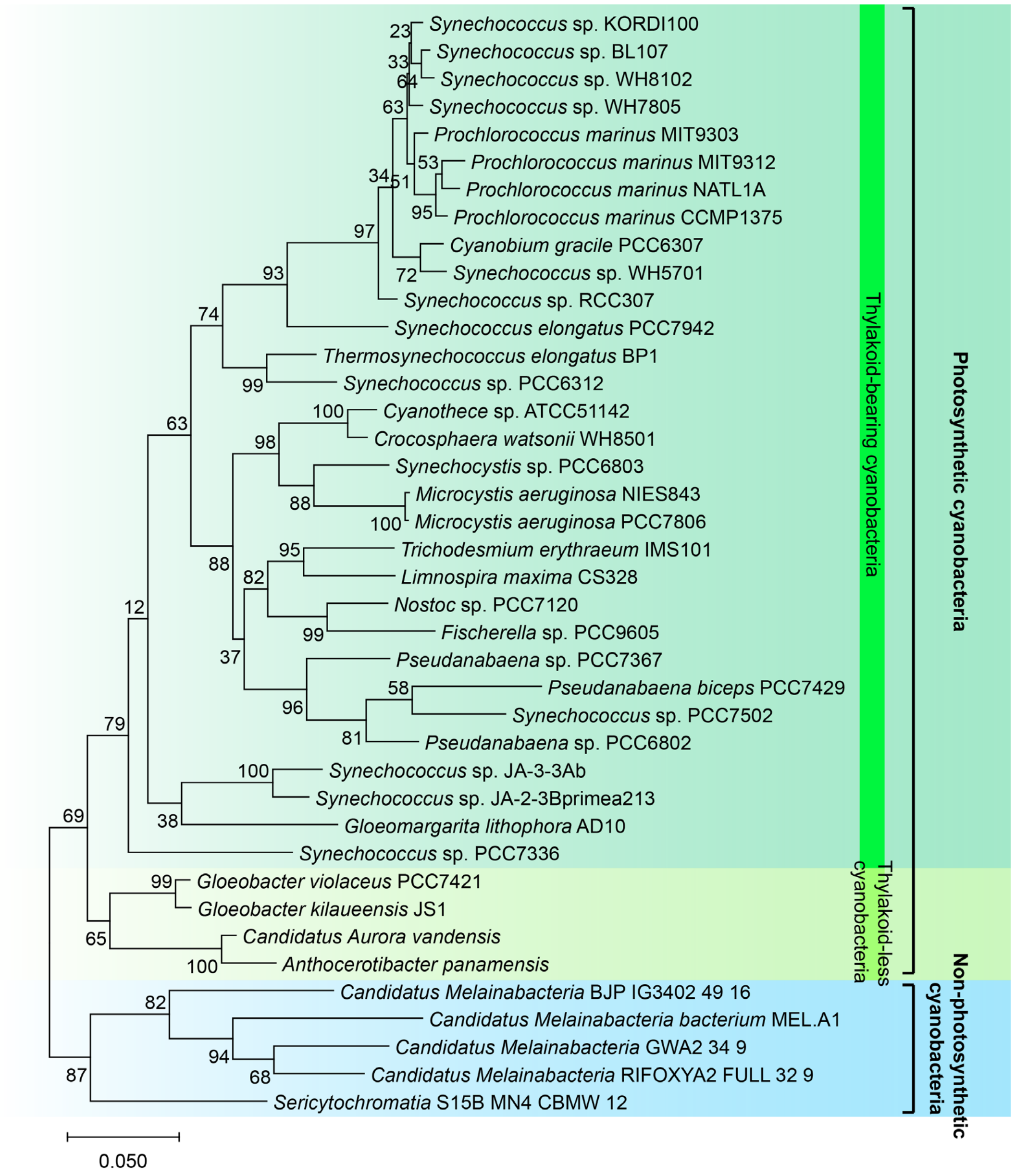

SUPPLEMENTARY FIGURE S2. Original phylogenetic tree of cyanobacteria used for time calibration. For details on cyanobacterial classification, refer to the legend of **Figure 1**.

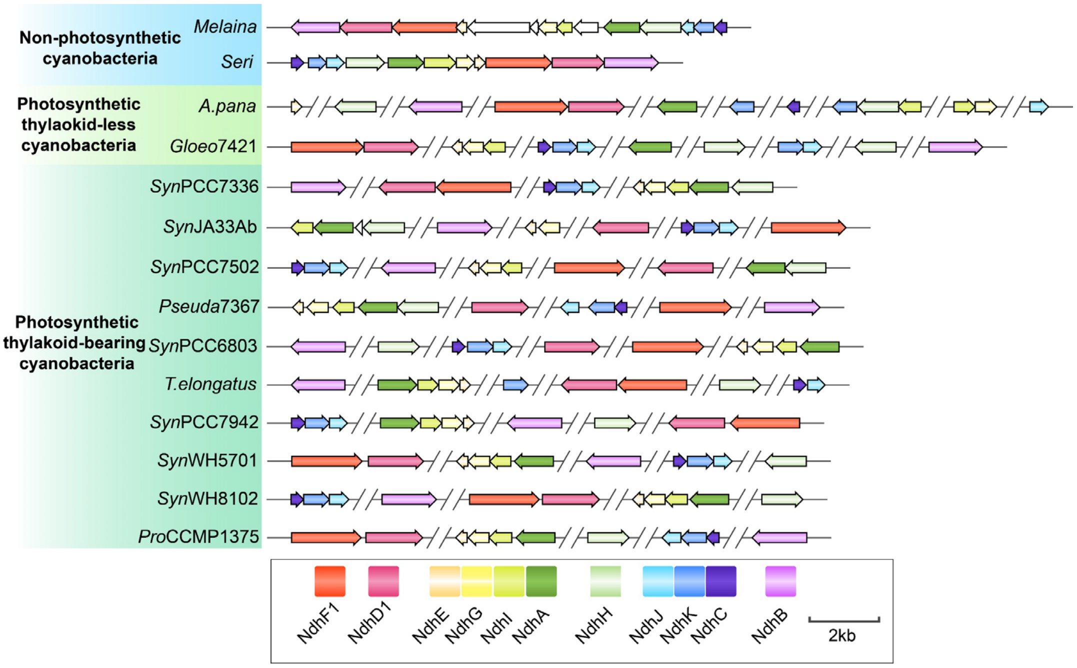

SUPPLEMENTARY FIGURE S3. Presence of conserved *ndh* genes in the phylum Cyanobacteria. The 11 conserved *ndh* genes are present across the Cyanobacteria phylum, encompassing both non-photosynthetic and photosynthetic species. Arrows, scaled to 2 kb, are color-coded to represent different *ndh* genes, while white arrows demote non-*ndh* gene. Species abbreviations are as follows: *Seri* (*Sericytochromatia*), *Melaina* (*Melainabacteria*), *A. pana* (*Anthocerotibacter panamensis*), *Gloeo*7421 (*Gloeobacter violaceus* PCC7421), *Syn*PCC7336 (*Synechococcus* sp. PCC7336), *Syn*JA33Ab (*Synechococcus* sp. JA-3-3Ab), *Syn*PCC7502 (*Synechococcus* sp. PCC7502), *Pseuda*7367 (*Pseudanabaena* sp. PCC7367), *Syn*PCC6803 (*Synechocystis* sp. PCC6803), *T. elongatus* (*Thermosynechococcus elongatus* BP-1), *Syn*PCC7942 (*Synechococcus* sp. PCC7942), *Syn*WH7501 (*Synechococcus* sp. WH5701), *Syn*WH8102(*Synechococcus* sp. WH8102), *Pro*CCMP1375 (*Prochlorococcus marinus* CCMP1375). The background colors used for species classification are consistent with those in **Figure 1**.

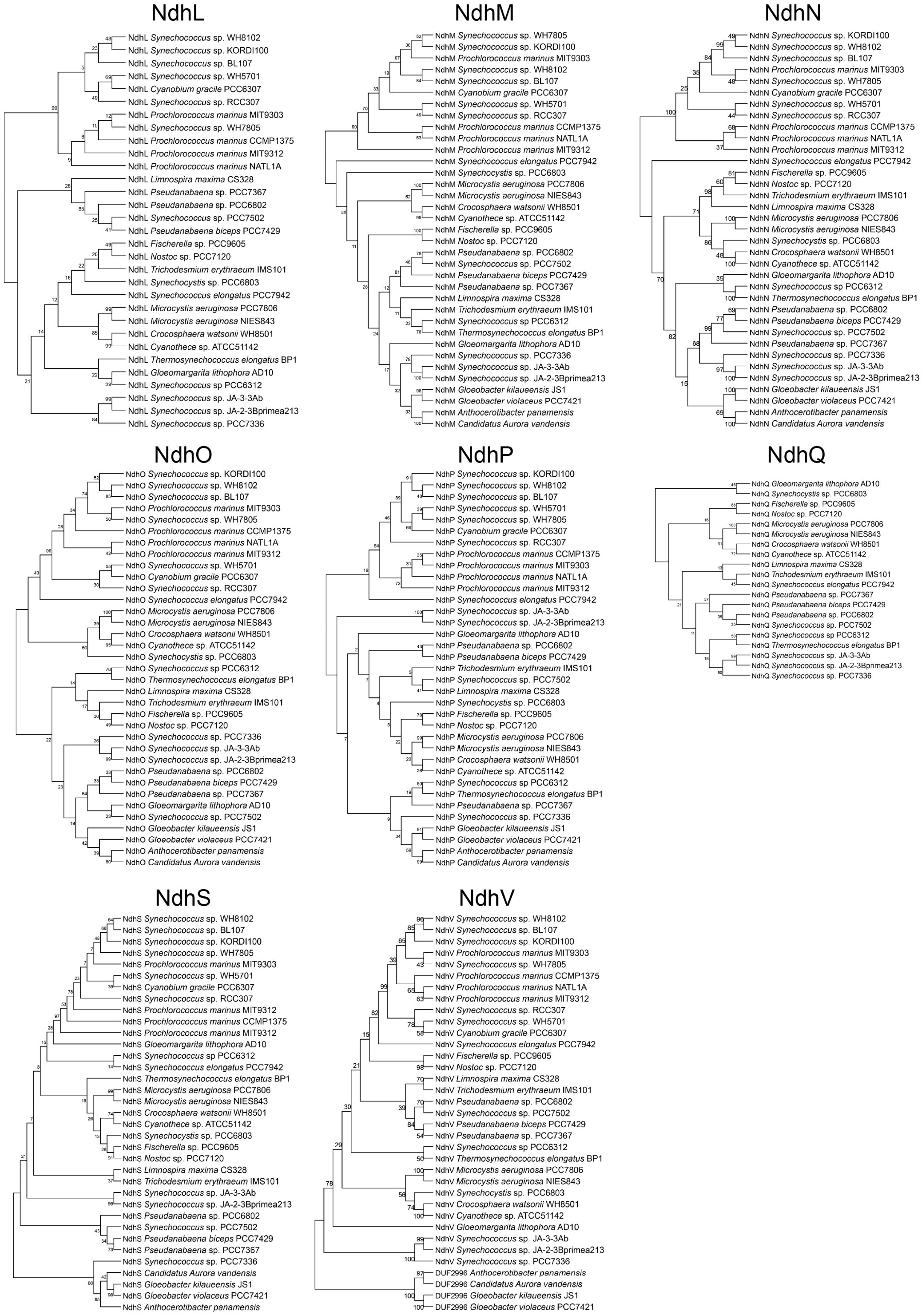

SUPPLEMENTARY FIGURE S4. Phylogenies of OPS subunits. Neighbor-joining phylogenetic trees of OPS subunits were constructed. Detailed sequences information is provided in **Supplementary Data 4**.

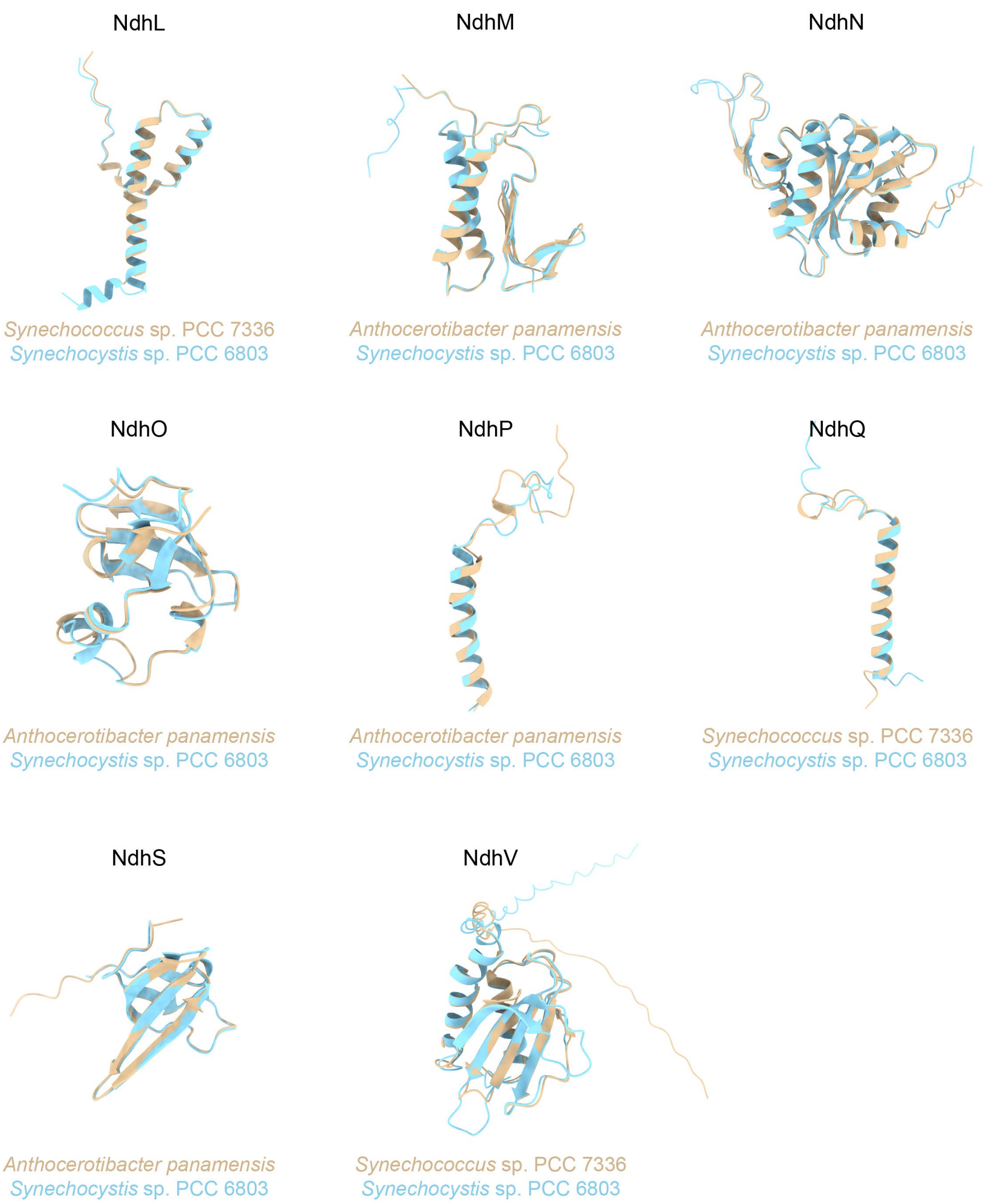

SUPPLEMENTARY FIGURE S5. Structural alignment of OPS subunits between *Synechocystis* sp. PCC6803 and its earliest homologs. The structures, predicted by AlphaFold 3, exhibit significant similarity, supporting further evidence for their homology. Detailed sequence information is available in **Supplementary Data 4**.

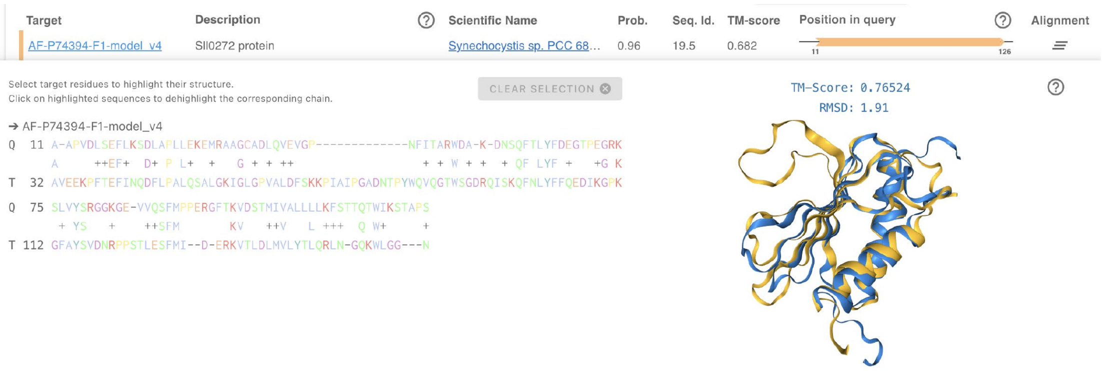

SUPPLEMENTARY FIGURE S6. Foldseek search identifies structural similarity between the DUF2996 domain-containing protein from *Anthocerotibacter panamensis* and NdhV from *Synechocystis* sp. PCC 6803. The predicted structure of DUF2996 domain-containing protein (WP_218080631.1) from *Anthocerotibacter panamensis* was used as a template for identifying structural homologs in *Synechocystis* sp. PCC 6803. The searching result suggest that NdhV (Sll0272) is the most likely homolog.
